# Genome characterization and phylogenetic analysis of the complete mitochondrial genome of *Eonemachilus caohaiensis* (Cypriniformes: Nemacheilidae)

**DOI:** 10.1080/23802359.2025.2602220

**Published:** 2025-12-15

**Authors:** Sheng Zeng, Xue Wang, Zhenyu Lv, Wei Wang, Jinli Hu, Xiaoping Zhang

**Affiliations:** ^a^ Guizhou Academy of Agriculture Sciences, Guizhou Fisheries Research Institute, Guiyang, Guizhou Province, P. R. China; ^b^ Guizhou Special Aquatic Products Engineering Technology Center, Guiyang, Guizhou Province, P. R. China

**Keywords:** *Yunnanilus*, mitochondrion, phylogeny, nemacheilidae

## Abstract

*Eonemachilus caohaiensis* is a bony fish endemic to Guizhou Province, China. In this study, we report its complete mitochondrial genome for the first time. The circular mitochondrial genome is 16,570 bp in length and consists of 13 protein-coding genes (PCGs), two rRNAs, 22 tRNAs, and a non-coding control region (D-loop). The overall nucleotide composition is A: 30.84%,T: 27.02%,C: 26.00%, and G: 16.14%. Maximum likelihood phylogeny positioned *E. caohaiensis* as a sister taxon to *Yunnanilus niger* and *E. longidorsali*, supporting their classification within the genus *Eonemachilus*. These findings provide new insights into the phylogenetic relationships within the Nemacheilidae family.

## Introduction

**Figure 1.**
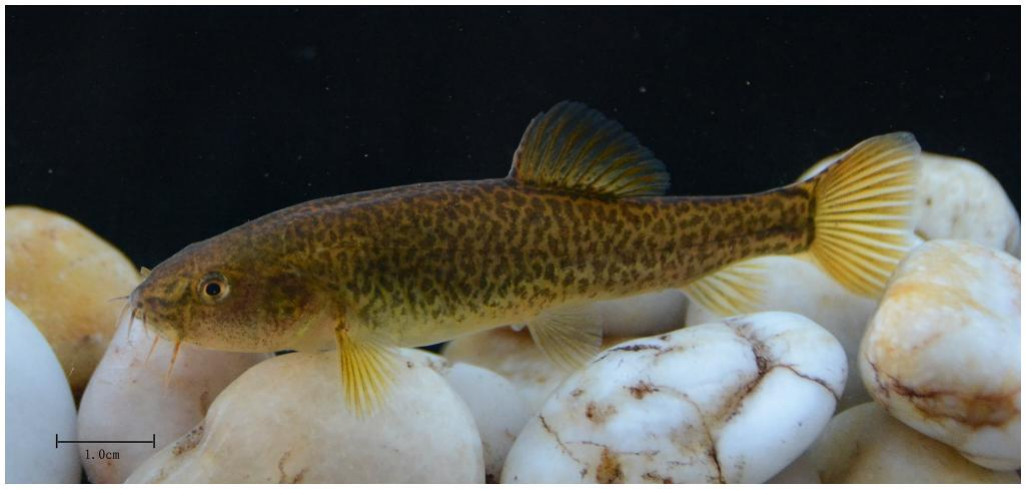
A reference image of *Eonemachilus caohaiensis* (body length:7.2 cm, body weight:5.5g) sequenced in this work, collected by Sheng Zeng, Wei Wang and Zhenyu Lv in Weining County (E 105°10′43″, N 26°43′9″), Guizhou Province, China. Photographed by Sheng Zeng at Weining County.

*Eonemachilus caohaiensis* (Figure 1), a narrow-range endemic loach of the Nemacheilidae family, is restricted to freshwater systems in Guizhou Province, China. Initially described from Caohai Lake (Weining County) (Ding 1992), the species was considered locally extirpated due to anthropogenic habitat degradation until its recent rediscovery in the Yangwanqiao River, a Luoze River tributary within the Yangtze River drainage (2022–2023 survey).

The Nemacheilidae is a large family comprising 792 species belonging to 49 genera (Fricke et al. 2025). Based on morphological characteristics, Prokofiev (2010) classified the Nemacheilidae family into five tribes (Lefuini, Nemacheilini, Triplophysini, Vaillantellini, and Yunnanilini). Among these, Vaillantellini is the most primitive group, forming sister group to the remaining four tribes; Lefuini and Yunnanilini share several primitive features. Yunnanilini originated slightly later than Lefuini; Triplophysini and Nemacheilini form a sister group, and together into Yunnanilini (Prokofiev, 2010; Du et al. 2021). species. Wang (2022) erected a new tribe Traccatichthyini, which containing the single genus Traccatichthys.

Berg first established the genus *Eonemachilus* (type species: N*emacheilus nigromaculatus*) without discussing its relationship with *Yunnanilus* (Berg 1938). Subsequent studies treated *Eonemachilus* as a synonym of *Yunnanilus* (Kottelat and Xin-Luo 1988; Yang 1991; Zhu 1989). Yang and Chen (1995) divided *Yunnanilus* into the *nigromaculatus* and *pleurotaenia* species groups based on the absence or presence of lateral line and cephalic lateral line canals, respectively. Kottelat (2012) later recognized *Eonemachilus* as a valid genus, including *E. nigromaculatus*, *E. yangzonghaiensis*, and *E. longidorsalis*, though without providing persuasive evidence. After examined 2490 specimens, Du et al. (2021) assigned the nigromaculatus species group (including *Eonemachilus obtusirostris*, *E. yangzonghaiensis, E. longidorsalis, E. caohaiensis, E. pachycephalus, E. nigromaculatus, E. bajiangensis, E. niger* and *E. altus*) to *Eonemachilus* based on anterior and posterior nostrils well-separated (distance larger than 1/2 of eye diameter), lateral line and cephalic lateral-line canals absent, and mouth terminal or inferior.

The taxonomic status of *E. caohaiensis* has been contentious. Initially described under *Yunnanilus* (Cobitidae) (Ding 1992), it was later transferred to *Eonemachilus* based on morphological synapomorphies such as separated nostrils and the absence of lateral-line pores (Du et al. 2021).

Here, we present the first complete mitochondrial genome of *E. caohaiensis* to clarify its systematic and phylogenetic position within the Nemacheilidae, evaluate the generic boundaries of *Eonemachilus* using mitogenomic data, and provide a genomic resource for the conservation of this rediscovered endemic species.

## Materials and methods

### Sample collection and preservation

A live adult specimen of *Eonemachilus caohaiensis* was collected from the Yangwanqiao River (26°43′09″N, 105°10′43″E), Weining County, Guizhou Province, China, during the 2022–2023 survey. Specimens were euthanized *via* MS-222 (tricaine methanesulfonate) immersion following AVMA guidelines. A voucher specimen (GZFRI20221129001) was fixed in 10% neutral buffered formalin for morphological analysis and subsequently transferred to 70% ethanol for long-term preservation at the Guizhou Fisheries Research Institute (Curator: Sheng Zeng; email: cloudzs@163.com). Pectoral fin tissue from a second live specimen was excised and preserved in 95% ethanol for molecular analysis.

### DNA extraction and sequencing

Total genomic DNA was extracted from the ethanol-preserved pectoral fin tissue using the Qiagen QIAamp Tissue Kit according to the manufacturer’s protocol. The complete mitochondrial genome was sequenced on the Illumina NovaSeq platform, generating 150-bp pair-end reads. Raw data were quality-filtered using Fastp v0.36 (Chen et al. 2018), yielding 58,990,260 clean pair-end reads, with a clean data rate of 99.456% and a Q30 bases ratio of 91.47%. The raw sequencing data were assembled using SPAdes (Bankevich et al. 2012). To validate the assembly accuracy, all filtered reads were remapped to the mitogenome, revealing high and uniform per-base sequencing depth which strongly supported the consistency of the final assembly with the raw data (Figures S1–S3). The mitochondrial genome structure was annotated using MitoFish (Iwasaki et al. 2013; Sato et al. 2018; Zhu et al. 2023), available at https://mitofish.aori.u-tokyo.ac.jp/. The annotated sequence has been submitted to GenBank under the accession number PP350837.

All experimental procedures complied to animal welfare guidelines and research regulations in China. The study protocol was approved by the Committee of Laboratory Animal Experimentation at the Guizhou Academy of Agricultural Sciences, Guizhou Fisheries Research Institute.

### Phylogenetic reconstruction

The phylogenetic tree was constructed using the mitogenome of *E. caohaiensis* and 29 other Nemacheilidae species as the ingroup. Species from the tribes Lefuini, Triplophysini, Traccatichthyini and Yunnanilini tribe were chosen due to their close relationships within Nemacheilidae (Prokofiev, 2010; Du et al. 2021, 2023). Two Cobitidae species, *Parabotia fasciata* and *Leptobotia elongata,* were designated as the outgroup. Complete mitogenome sequences were aligned using the MUSCLE in MEGA12 with default settings (Kumar et al. 2024).

**Figure 2.**
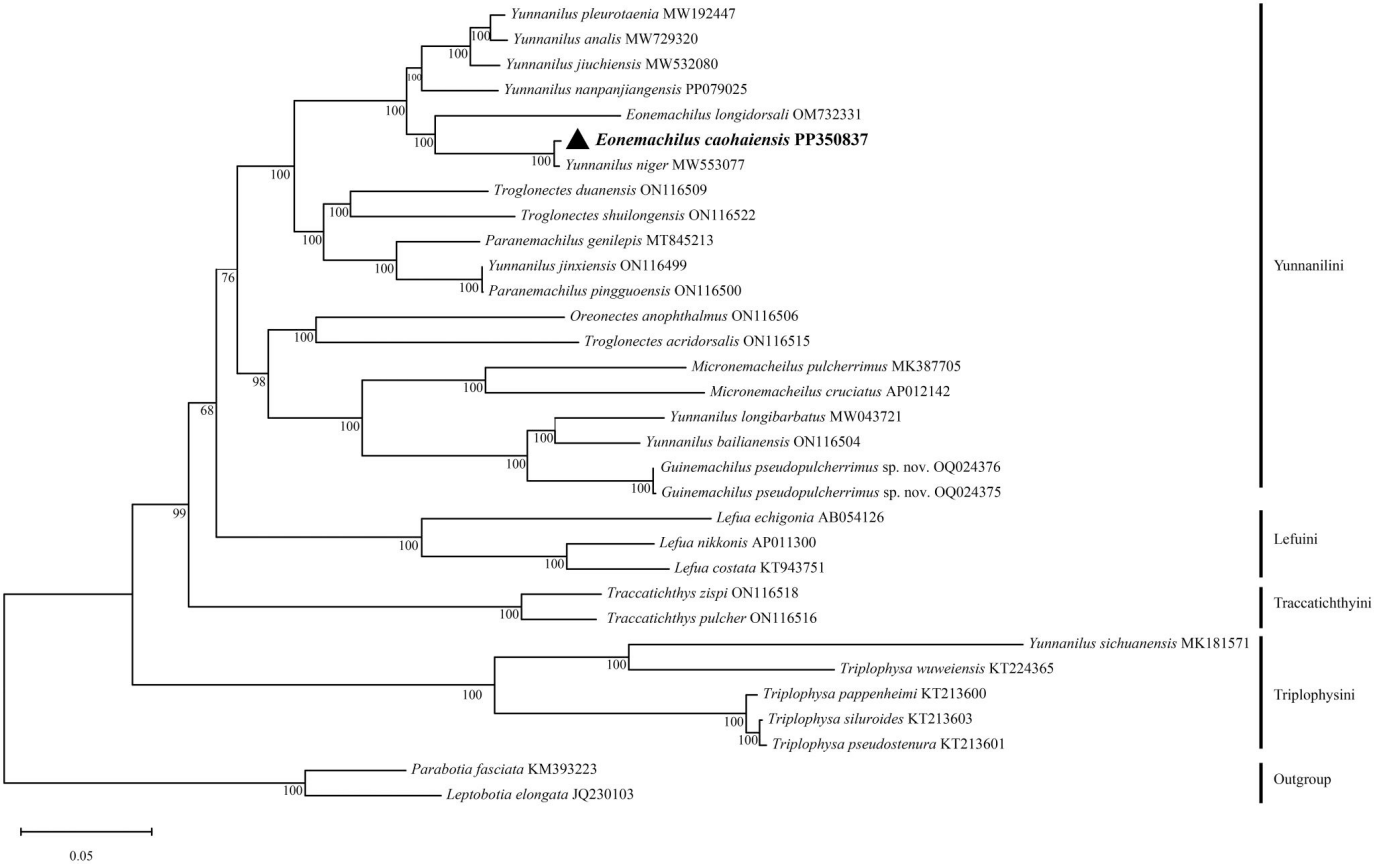
Phylogenetic relationships between *Eonemachilus caohaiensis* (PP350837) and other Nemacheilidae species based on complete mitochondrial genome sequences. *Parabotia fasciata* (KM393223) and *Leptobotia elongata* (JQ230103) were chosen as the outgroups. Numbers under the nodes are bootstrap support for maximum likelihood. The following sequence were used: *Eonemachilus longidorsali* OM732331 (Song, Direct Submission), *Triplophysa wuweiensis* KT224365 (Wang et al. 2016), *Triplophysa pseudostenura* KT213601 (Wang et al. 2016), *Triplophysa pappenheimi* KT213600 (Wang et al. 2016), *Triplophysa siluroides* KT213603 (Wang et al. 2016), *Paranemachilus pingguoensis* ON116500 (Luo et al. 2023), *Paranemachilus genilepis* MT845213 (Luo et al. 2020), *Yunnanilus pleurotaenia* MW192447. (Cui et al. Direct Submission), *Yunnanilus niger* MW553077 (Cui et al. Direct Submission), *Yunnanilus jiuchiensis* MW532080 (Du et al. 2021), *Yunnanilus jinxiensis* ON116499 (Luo et al. 2023), *Yunnanilus bailianensis* ON116504 (Luo et al. 2023), *Yunnanilus analis* MW729320 (Qiu and Chen, Direct Submission), *Yunnanilus nanpanjiangensis* PP079025 (Wu and Lei, Direct Submission), *Yunnanilus sichuanensis* MK181571 (Zou et al. 2019), *Yunnanilus longibarbatus* MW043721 (Zhou, Direct Submission), *Micronemacheilus cruciatus* AP012142, (Miya, Direct Submission), *Micronemacheilus pulcherrimus* MK387705 (Luo et al. 2019), *Guinemachilus pseudopulcherrimus* sp. nov. OQ024376 and OQ024375 (Du et al. 2023), *Troglonectes acridorsalis* ON116515 (Luo et al. 2023), *Oreonectes anophthalmus* ON116506 (Luo et al. 2023), *Traccatichthys zispi* ON116518 (Luo et al. 2023), *Traccatichthys pulcher* ON116516 (Luo et al. 2023), *Lefua echigonia* AB054126 (Saitoh et al. 2003), *Lefua nikkonis* AP011300 (Miya et al. 2015), *Lefua costata* KT943751 (Park et al. Direct Submission), *Troglonectes duanensis* ON116509 (Luo et al. 2023), *Troglonectes shuilongensis* ON116522 (Luo et al. 2023).

The phylogeny was inferred using the Maximum Likelihood method. The best-fit substitution model (GTR+G + I) was selected based on the Akaike Information Criterion (AIC) in jModeltest v2.1.10 (Darriba et al. 2012). The tree with the highest log likelihood (−132044.35) is shown (Figure 2). The percentage of replicate trees in which the associated taxa clustered together (1,000 replicates) is shown below the branches (Felsenstein 1985). Initial tree(s) for the heuristic search were obtained automatically by applying the Maximum Parsimony method. The evolutionary rate differences among sites were modeled using a discrete Gamma distribution across 5 categories (+G, parameter = 1.0640), with 54.07% of sites deemed evolutionarily invariant (+I). The analytical procedure encompassed 32 nucleotide sequences with 16,904 positions in the final dataset. All evolutionary analyses were conducted in MEGA12.

## Results

**Figure 3.**
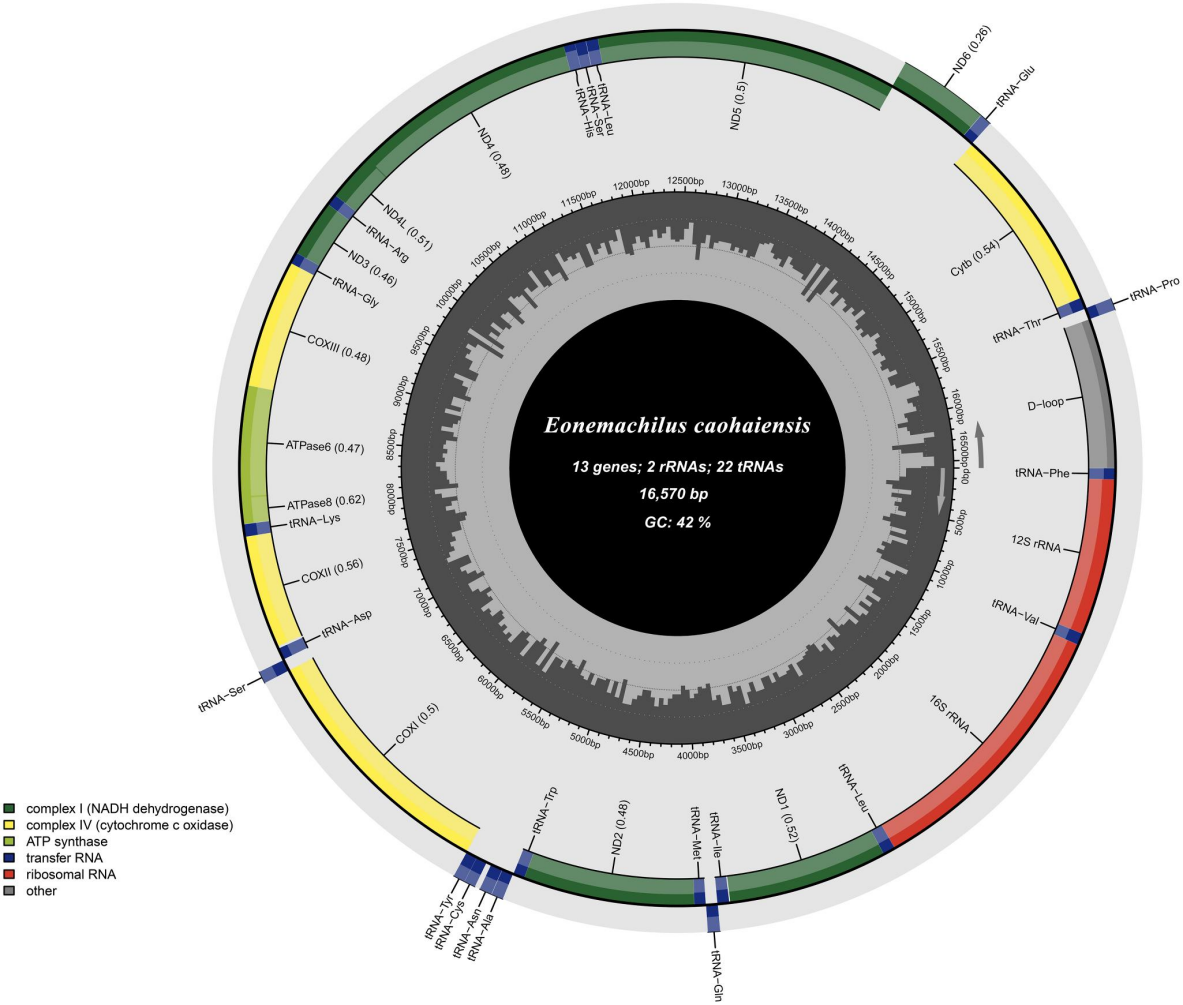
Organization of the sequenced mitochondrial genome of *Eonemachilus caohaiensis.* The genes scattered on the heavy strand are shown on the outer side of the circle, while the inner sideshows those that are scattered on the light strand.

The mitochondrial genome of *E. caohaiensis* is a circular molecule 16,570 bp in length, consistent with the typical structure observed in other vertebrates. It contains 13 protein-coding genes(PCGs), two ribosomal RNA (rRNA) genes, 22 transfer RNA (tRNA) genes, and a non-coding control region (D-loop) (Figure 3). The mitogenome is AT skewed (57.86% A + T), with nucleotide frequencies of 30.84% A, 27.02% T, 26.00% C, and 16.14% G. With the exception of one protein-coding gene (*ND6*) and eight tRNA genes (*trnQ*, *trnA*, *trnN*, *trnC*, *trnY*, *trnS*, *trnE*, and *trnP*) encoded on the L-strand, all other genes were located on the H-strand.

Among the 13 PCGs, most started with the standard start codon ATG, except for *COX1*, which uses GTG-a pattern commonly observed in other vertebrates. Seven genes terminated with complete stop codons, (TAG for *ND1*; TAA for *ND5*, *ND6*, *COX1*, *ATP6*, *ATP8*, and *ND4L*), while the remaining genes have incomplete stop codons, either T (*COX2*, *COX3*, *ND2*, *ND3*, and *CYTB*) or TA (*ND4*). The D-loop region, spanning 915 bp, was located between *trnP* and *trnF*.

*Eonemachilus caohaiensis* was fully resolved (BS = 100) with *Yunnanilus niger*. These two were positioned in a clade with strong support (BS = 100) with *E. longidorsali*. A sister clade containing *Y. jiuchiensis*, *Y. pleurotaenia*, *Y. analis*, and *Y. nanpanjiangensis* was inferred. However, the genus *Yunnanilus* appears polyphyletic in our analysis: *Y. jinxiensis* and *Y. sichuanensis* grouped with species from the genera *Paranemachilus* and *Triplophysa*, respectively, while *Y. longibarbatus* and *Y. bailianensis* clustered with species from *Guinemachilus* (Figure 2).

## Discussion and conclusion

We assembled the first complete mitochondrial genome of *Eonemachilus caohaiensis* (16,570 bp), providing definitive genomic evidence for its taxonomic reclassification. After examined mutiple specimens, Du et al. (2021) assigned *E. caohaiensis* (whole body covered with scales; pectoral fin with 10–11 branched rays; caudal fin with 14 branched rays; outer gill rakers on first gill arch absent, 9–13 inner gill rakers on first gill arch; processus dentiformis present; body depth 22.1%–29.9% standard length) to *Eonemachilus,* along with other members of the *nigromaculatus* species group, based on shared morphological features. Although the complete mitogenome of *E. nigromaculatus* (the generitype of *Eonemachilus*) was not included in our phylogenomic analysis due to tissue misidentified issues (Du et al. 2023), our phylogenomic results robustly supports the clustering of *E. caohaiensis* with *E. longidorsalis* and *Yunnanilus niger*, corroborating Du et al. (2021) morphological hypothesis that these species are congeneric within *Eonemachilus*. Both morphological and molecular evidence support the recognition of *Eonemachilus* as a distinct genus.

Notably, *Yunnanilus jinxiensis, Y. sichuanensis*, *Y. longibarbatus* and *Y. bailianensis* did not cluster with other *Yunnanilus* species in the phylogenetic tree, suggesting that these four species may belong to different genera within Nemacheilidae. Specifically, *Y. jinxiensis and Y. sichuanensis* may belong to *Paranemachilus* and *Triplophysa* respectively, while *Y. longibarbatus* and *Y. bailianensis* may represent a new genus, *Guinemachilus* (Du et al. 2023).

This study offers valuable insights into the phylogenetic relationships within Nemacheilidae, enhancing our understanding of the evolutionary history and taxonomic structure of this diverse fish family.

## Supplementary Material

Supplemental material.docx

## Data Availability

The genome sequence data that support the findings of this study are openly available in GenBank of NCBI at (https://www.ncbi.nlm.nih.gov/) under the accession no. PP350837. The associated BioProject, Bio-Sample and SRA numbers are PRJNA1077888, SAMN39983804 and SRR28248341 respectively.

## Vulnerable species statement

We have strictly followed the policies of the International Union for Conservation of Nature (IUCN) policies research involving species at risk of extinction, the Convention on Biological Diversity and the Convention on the Trade in Endangered Species of Wild Fauna and Flora.
