# Supplementary material for: Genome characterization and phylogenetic analysis of the complete mitochondrial genome of Eonemachilus caohaiensis (Cypriniformes: Nemacheilidae)
Source: Mitochondrial DNA B Resour. 2025 Dec 15;11(1):54–8. doi: 10.1080/23802359.2025.2602220 (PMC12707081; doi:10.1080/23802359.2025.2602220)
Supplement: Supplemental material.docx [file TMDN_A_2602220_SM7963.docx]

**Genome characterization and phylogenetic analysis of the complete mitochondrial genome of** ***Eonemachilus caohaiensis* (Cypriniformes: Nemacheilidae)**

Sheng Zeng^a,b^, Xue Wang^a,b^, Zhenyu Lv^a,b^, Wei wang^a,b^,Jinli Hu^a,b^, Xiaoping Zhang^a,b^

^a^Guizhou Academy of Agriculture Sciences, Guizhou Fisheries Research Institute, Guiyang 550025 Guizhou Province, P. R. China; ^b^ Guizhou special aquatic products engineering technology center, Guiyang 550025 Guizhou Province, P. R. China.


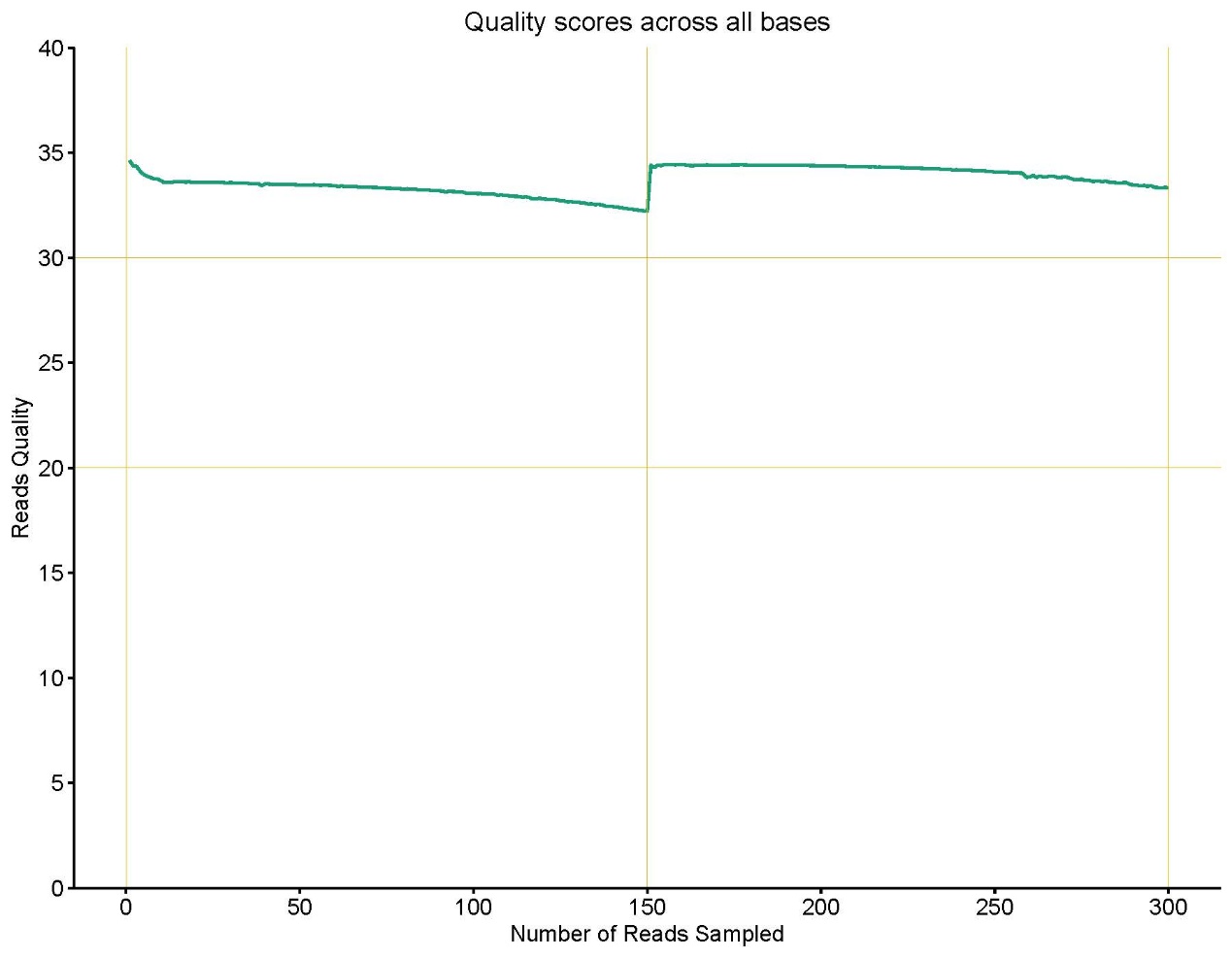


Figure S1 Per base sequence quality. The horizontal axis represents the base position of reads, and the vertical axis represents the average sequencing quality of all reads at that position. The left side of the dash line from 0 to 150bp represents the sequencing quality distribution of read-1, and the right side from 150 to 300bp represents the sequencing quality distribution of read-2.


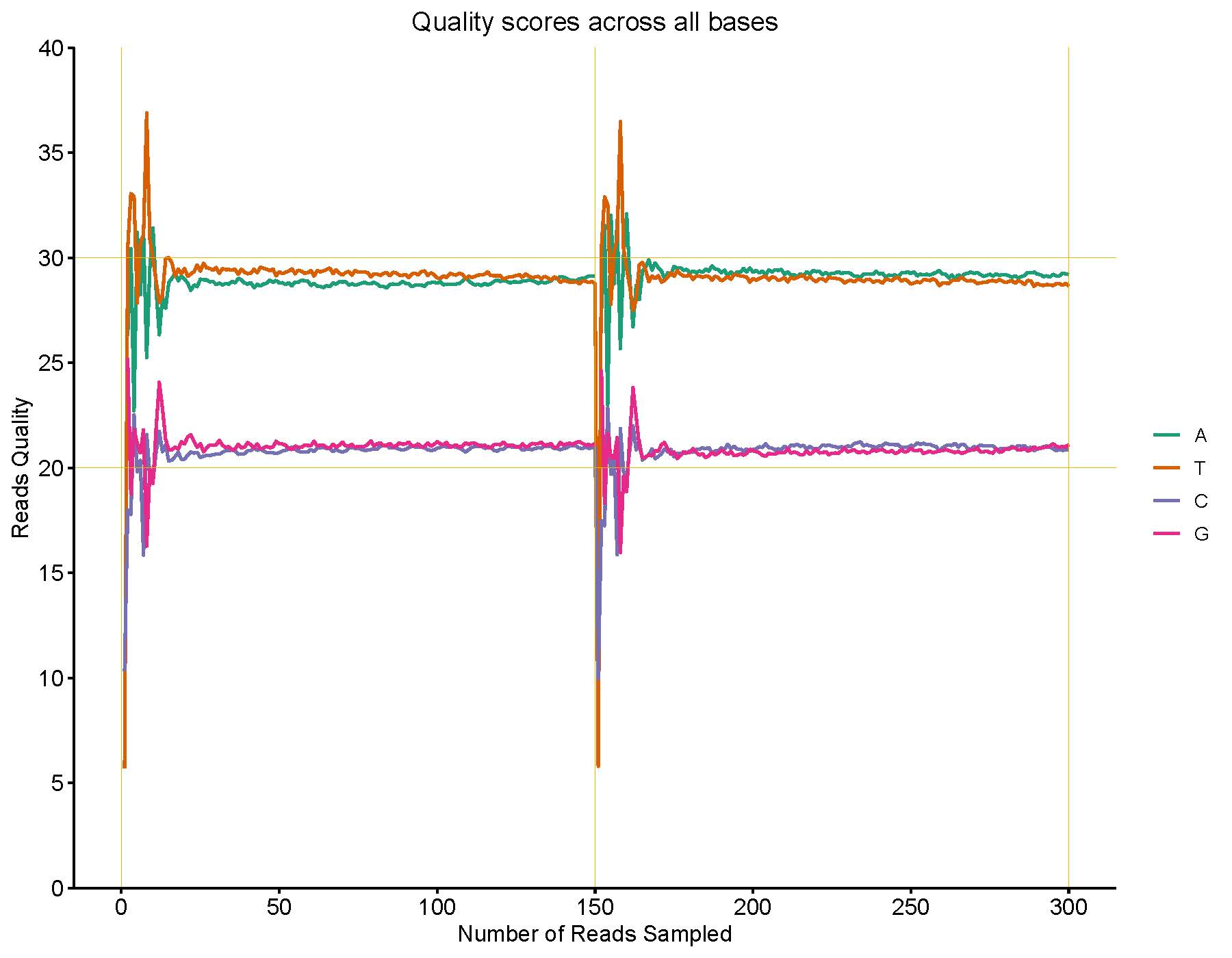


Figure S2 Per base sequence content. The horizontal axis represents the base position of the reads, and the vertical axis represents the content of four nucleotides (ATGC) at that position for all reads. The left side of the dashed line from 0 to 150bp represents read-1, and the right side from 150 to 300bp represents read-2.


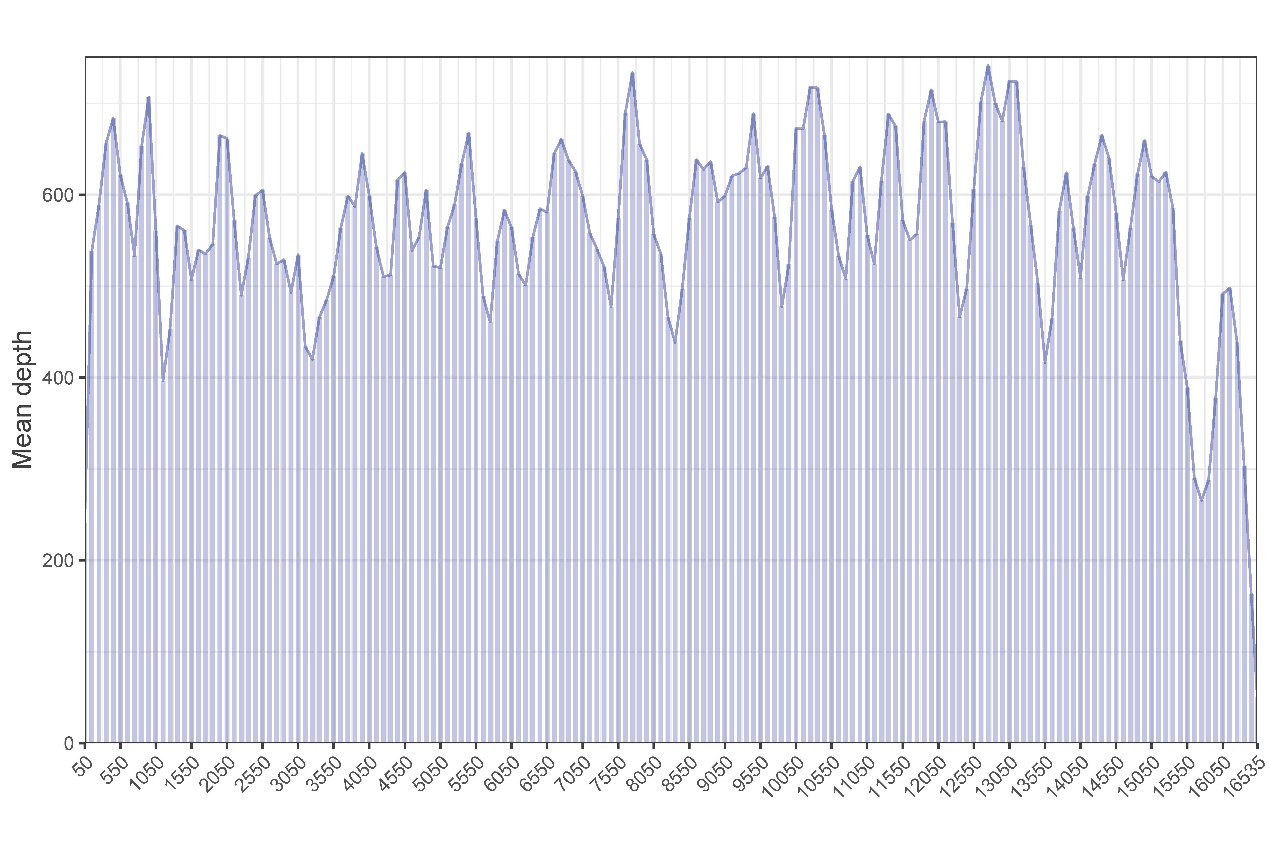


FigureS3 Sequencing Depth and Coverage map of the mitochondrial genome of *Eonemachilus caohaiensis.* The final 16,570 bp assembly exhibits uniformly high coverage across the genome, with a mean depth of 462.69.

Table S1

| Total Reads Count(#) | 59550228 |
| --- | --- |
| Total Bases Count(bp) | 8932534200 |
| Average Read Length(bp) | 150 |
| Q20 Bases Count(bp) | 8664717847 |
| Q20 Bases Ratio(%) | 97.00% |
| Q30 Bases Count(bp) | 8169332982 |
| Q30 Bases Ratio(%) | 91.46% |
| GC content(%) | 41.82% |

|  | Read1 | Read2 |
| --- | --- | --- |
| Total Reads Count(#) | 29775114 | 29775114 |
| Total Bases Count(bp) | 4466267100 | 4466267100 |
| Average Read Length(bp) | 150 | 150 |
| Q20 Bases Count(bp) | 4284272553 | 4380445294 |
| Q20 Bases Ratio(%) | 95.93% | 98.08% |
| Q30 Bases Count(bp) | 3955451106 | 4213881876 |
| Q30 Bases Ratio(%) | 88.56% | 94.35% |
